# FADS Gene Polymorphisms Confer the Risk of Coronary Artery Disease in a Chinese Han Population through the Altered Desaturase Activities: Based on High-Resolution Melting Analysis

**DOI:** 10.1371/journal.pone.0055869

**Published:** 2013-01-31

**Authors:** Si-Wei Li, Kun Lin, Pei Ma, Zhen-Lu Zhang, Yi-Dan Zhou, Shuang-Yan Lu, Xin Zhou, Song-Mei Liu

**Affiliations:** 1 Center for Gene Diagnosis, Zhongnan Hospital of Wuhan University, Wuhan, Hubei, People's Republic of China; 2 Department of Cardiology, Wuhan Asia Heart Hospital, Wuhan, Hubei, People's Republic of China; 3 School of Life Sciences, Jianghan University, Wuhan, Hubei, People's Republic of China; College of Pharmacy, University of Florida, United States of America

## Abstract

**Objective:**

We explored the desaturase activities and the correlation of fatty acid desaturases (FADS) gene single nucleotide polymorphisms (SNPs) with plasma fatty acid in coronary artery disease (CAD) patients in a Chinese Han population.

**Methods:**

Plasma fatty acids were measured by gas chromatography in CAD patients (n = 505) and a control group (n = 510). Five SNPs in the FADS gene were genotyped with high-resolution melting (HRM) methods.

**Results:**

After adjustment, D6D activity, assessed as arachidonic acid (AA, C20:4n-6)/linoleic acid (LA, C18:2n-6), was higher in CAD patients (p<0.001). D9D activity, which was estimated as the ratio of palmitoleic acid (C16:1)/palmitic acid (C16:0) or oleic acid (C18:1n-9) to stearic acid (C18:0), was also increased (p<0.001). The genotype distributions of rs174537 G>T and rs174460 C>T were different between the two groups. The rs174537 T allele was associated with a lower risk of CAD [OR 0.743, 95% CI (0.624, 0.884), p = 0.001]. Carriers of the rs174460 C allele were associated with a higher risk of CAD [OR 1.357, 95% CI (1.106, 1.665), p = 0.003].

**Conclusions:**

We firstly report that the rs174460 C allele is associated with a higher risk of CAD, and confirm that the rs174537 T allele is associated with a lower risk of CAD. Our results indicate that FADS gene polymorphisms are likely to influence plasma fatty acid concentrations and desaturase activities.

## Introduction

Coronary artery disease (CAD), the major type of cardiovascular disease, is becoming the number one cause of morbidity and mortality among adults in China. Dyslipidemia, diabetes mellitus, hypertension, obesity, and fatty acid metabolic abnormalities are risk factors for the development of atherosclerosis [1]. In recent years, fatty acid "lipotoxicity" has gradually become a new research point. Studies have shown that saturated fatty acids can promote the risk of CAD, while unsaturated fatty acids reduced this risk [1], [2]. Polyunsaturated fatty acids (PUFAs) are also processed to powerful promoters of inflammation. Recent reports have highlighted the influence of fatty acid composition on metabolic syndrome and arterial stiffness, such as linoleic acid (LA, C18:2n-6) and dihomo-γ-linolenic acid (DGLA, C20:3n-6) [1], [3]. On the contrary, some findings suggested that sufficient amounts of LA can reduce cardiovascular risk [3], [4]. Dietary eicosapentaenoic acid (EPA, C20:5n-3) and docosahexaenoic acid (DHA, C22:6n-3) can prevent sudden cardiac death, acute coronary syndrome, and heart failure [5].

The concentration of plasma fatty acids is influenced by dietary intake and metabolic pathways [4]. Fatty acid desaturases (FADS) are key enzymes in fatty acid metabolism. In humans, there are three main desaturases: stearoyl-CoA desaturase (SCD), also known as delta-9-desaturase (D9D), catalyzes the synthesis of monounsaturated fatty acids; delta-5-desaturase (D5D) catalyzes the synthesis of highly unsaturated fatty acids (HUFAs) and delta-6-desaturase (D6D) is required in the synthesis of HUFAs. D9D, encoded by the SCD gene, has recently been found as an independent risk factor of cardiovascular disease [6]. D5D and D6D are encoded by fatty acid desaturase 1 (FADS1) and fatty acid desaturase 2 (FADS2) genes, respectively. Recently, several studies have shown a strong association between plasma PUFAs and FADS gene polymorphisms [7], [8]. Plasma arachidonic acid (AA, C20:4n-6) and EPA concentrations have been confirmed to be associated with rs174537 near FADS1 gene by a GWAS [9]. Merino DM et al. recently reported that polymorphisms of rs174547 in FADS1 gene and rs498793 in FADS2 gene alter desaturase activity in young Caucasian and Asian adults [10]. The minor allele of rs174537, rs174545, rs174546, rs174553, rs174556, rs174561, rs174568, rs99780, rs174570, rs174575, rs2524299, rs174583, rs498793, rs174611, rs174627 and rs1000778 in FADS gene was consistently associated with decreased n-6 PUFAs, with the exception of DGLA [11]. However, research on association between FADS genes polymorphisms and fatty acid concentrations in the Chinese population is still rare.

High-resolution melting (HRM) analysis is a relatively new and rapid method to detect single nucleotide polymorphisms (SNPs). It monitors the PCR process by saturated fluorescent dye-LC Green. Different bases cause change in annealing temperature and leading to the formation of different melting curves in the heating process [12]. Thus different SNP loci, heterozygous and homozygous can be effectively distinguished.

Therefore, this study aims to explore the desaturase activities and the correlation of FADS gene SNPs with plasma fatty acids in CAD patients in a Chinese Han population.

## Materials and Methods

### Study population and blood collection

CAD patients without cancer or diabetes were recruited from Wuhan Asia Heart Hospital. CAD was defined as follows: (1) angiography showed ≥50% stenosis in one or more major coronary artery; (2) myocardial infarction diagnosis according to the WHO criteria issued in 1979 [13], including clinical symptoms, enzyme elevation or ECG changes; (3) absence of atherosclerosis, or occlusion of vascular stenosis and spasm; and (4) no clinical or pathological changes or any diagnosis of the diseases mentioned above. Control participants were recruited from Zhongnan Hospital of Wuhan University. Finally, 1015 genetically unrelated Chinese subjects (33–85 years) were included in this study (505 CAD, 510 controls). The participation rates in case and control subjects from recruitment were 49.8% and 50.2%, respectively. Written informed consent was obtained from each participant, and the study protocol was approved by the ethics committees of Zhongnan Hospital of Wuhan University and Asia Heart Hospital. After an overnight fast, samples of venous blood were drawn from each subject into EDTA tubes. The tubes were immediately placed on ice until they arrived at the laboratory. Then, the blood specimens were separated into plasma, and stored at −80°C until analysis.

### Measurement of fatty acid levels and desaturase activity

The fatty acids were extracted from 200 µl of plasma and converted into their methyl esters by transesterification using the methods described previously [14].The fatty acid methyl esters were analyzed using gas chromatography (Varian 450-GC, varian Inc., USA) on a 10 m×0.1 mm×0.1 µm polyethylene glycol column (DB-WAX, Agilent Technologies, USA). Peaks were identified by comparison with fatty acid methyl ester standards (Sigma-Aldrich, USA) using a mass spectrometer (Varian 320-MS TQ Mass spectrometer, varian Inc., USA). The concentration of each fatty acid was expressed as a percentage of total fatty acids. D5D activity was estimated as the ratio of AA to DGLA. D6D activity was estimated as the ratio of AA to LA [15]. D9D activity was estimated as the ratio of palmitoleic acid (C16:1) to palmitic acid (C16:0) for D9D-16 and the ratio of oleic acid (C18:1n-9) to stearic acid (C18:0) for D9D-18. All of the coefficients of variance were less than 5%.

### Single-nucleotide polymorphisms and genotyping

Five SNPs showing the greatest variability in the FADS gene region were selected from the HapMap database with the criteria used in our SNP selection procedure [a minor allele frequency over 0.1 and tag SNPs with an r^2^ value above 0.8]. Rs174537 was from block 1, rs174550 was from block 2, and rs174460 was from block 3. Rs174611 and rs174616 were between blocks 1 and 2. Table 1 presents a description of the SNPs used. Genomic DNA was extracted from 200 µl whole blood using sodium iodide extraction technique. SNPs were genotyped by high-resolution melting of small amplicons on LightScanner 32 instrument (Idaho Technology, USA). Primer details and product lengths are shown in Table S1.

**Table 1:** Characteristics of SNPs in FADS gene cluster.

SNP	Gene	Position*^1^*	Minor allele	Major allele	MAF*^2^*
rs174537	near FADS1	61552680	T	G	0.333
rs174616	FADS2	61629122	T	C	0.454
rs174611	FADS2	61627881	C	T	0.143
rs174460	FADS3	61657110	C	T	0.474
rs174450	FADS3	61641542	C	A	0.462

*1*: Position in basepairs was derived from dbSNP Build 137. Based on NCBI Human Genome Build 37.3 (November, 2012) of chromosome 11.

*2*: MAF, minor allele frequency.

### Statistical methods

Statistical analyses were performed with SPSS 13.0 for Windows. Normally distributed data were shown as the mean±S.D. Skewed data were described by the median and interquartile range. When a normal distribution and equal variance were found between the two groups, a t-test was used. When equal variances were not assumed, a t′-test was used. Skewed data were log transformed before analysis to achieve a normal distribution. Hardy-Weinberg Equilibrium, genotype and allele frequency distributions were analyzed by the chi-square test. Logistic regression analysis was also applied, with adjustment for confounders. Haploview software was used to evaluate the linkage disequilibrium of SNPs. One-way ANOVA analysis or K-Independent non-parametric analysis was used to compare the fatty acid levels among the genotype groups. P values less than 0.05 were considered statistically significant.

## Results

### General characteristics, plasma fatty acids and desaturase activity of the control and CAD patients

The general characteristics of the control and CAD patients are summarized in Table 2. Except for gender, age and diastolic, all the characteristics were different between the two groups (p<0.01). Figure S1 shows the Chromatograms of plasma fatty acids. The plasma fatty acid concentration differed between controls and CAD patients in several instances (Table 3). After adjustment for gender, age, body mass index (BMI), blood pressure, Total-cholesterol (TC), Triglyceride (TG), HDL-cholesterol (HDL-C) and LDL-cholesterol (LDL-C), CAD patients had higher concentrations of C16:0, C16:1, C18:1n-9, AA, total monounsaturated fatty acids, and lower concentrations of LA, DHA, as well as total polyunsaturated n-3 and n-6 fatty acids. As a consequence, D6D activity, presented as AA/LA, was higher in CAD patients (p<0.001). D9D activities, estimated as the ratio of both C16:1/C16:0 and C18:1n-9/C18:0, were all increased in CAD patients (p<0.001). No significant difference in D5D activity (AA/DGLA) or n-3/n-6 was found between control and CAD patients.

**Table 2:** Characteristics of controls and CAD patients.

Characteristics	Controls (n = 510)	CAD patients (n = 505)	P
Male/Female (%)	59.4/40.6	55.0/45.0	0.160
Age(year)*^1^*	59.09±9.47	59.45±9.69	0.496
BMI(kg/m^2^)*^1^*	23.5±3.3	25.9±3.1	<0.001
Systolic(mmHg)*^1^*	126.6±17.3	129.7±16.62	<0.001
Diastolic(mmHg)*^1^*	77.2±8.9	76.9±10.1	0.091
TC(mmol/l)*^2^*	4.46(3.98, 4.89)	4.05(3.32, 4.77)	<0.001
TG(mmol/l)*^2^*	1.04(0.79, 1.36)	1.3(0.97, 1.70)	<0.001
HDL-C(mmol/l)*^2^*	1.3(1.12, 1.51)	1.14(0.95, 1.34)	<0.001
LDL-C (mmol/l)*^2^*	2.75(2.35, 3.05)	2.42(1.81, 2.93)	<0.001
FPG (mmol/l)*^2^*	4.92(4.60, 5.32)	5.84(5.22, 6.38)	<0.001

BMI: body mass index, TC: Total-cholesterol, TG: Triglyceride, HDL-C: HDL-cholesterol, LDL-C: LDL-cholesterol, FPG: Fasting plasma glucose.

*1*: Mean±SD.

*2*: Median (25 Percentiles, 75 Percentiles).

**Table 3:** Plasma fatty acid composition and desaturase activity of controls and CAD patients.

Characteristics	Controls (n = 510)	CAD patients (n = 505)	P*^4^*
Total saturated fatty acid*^1,2^*	31.64±4.88	32.00±4.54	0.235
Palmitic acid, C16:0*^1,2^*	22.47±3.43	22.98±3.39	0.038
Stearic acid, C18:0*^1,2^*	9.17±1.82	9.02±1.64	0.386
Total monounsaturated fatty acid*^1^*	15.65±3.22	17.31±3.60	<0.001
Palmitoleic acid, C16:1*^3^*	0.69(0.50, 0.95)	0.93(0.65, 1.24)	<0.001
Oleic acid, C18:1n-9*^1^*	14.89±3.01	16.26±3.19	<0.001
Total polyunsaturated n-3 fatty acid*^3^*	3.58(2.93, 4.33)	3.42(2.83, 4.03)	0.003
α -linolenic acid, C18:3n-3*^3^*	0.52(0.33, 0.75)	0.57(0.34, 0.79)	0.428
Eicosapentaenoic acid, C20:5n-3*^3^*	0.20(0.00, 0.44)	0.17(0.00, 0.40)	0.065
Docosahexaenoic acid , C22:6n-3*^1^*	2.79±0.95	2.55±0.88	0.001
Total polyunsaturated n-6 fatty acid*^3^*	46.93(43.84, 50.06)	44.06(41.49,47.55)	<0.001
Linoleic acid, C18:2n-6*^1,2^*	35.62±6.93	32.66±6.40	<0.001
γ-linolenic acid, C18:3n-6*^3^*	0.20(0.03, 0.39)	0.30(0.09, 0.55)	0.645
Dihomo-γ-linolenic acid, C20:3n-6*^3^*	1.33(1.00, 1.65)	1.55(1.16, 2.04)	0.307
Arachidonic acid, C20:4n-6*^1^*	7.82±2.21	8.15±2.64	0.004
Desaturase activity			
C20:4n-6/C20:3n-6 (D5D)*^3^*	6.15(4.50, 7.93)	5.28(3.51, 7.70)	0.699
C20:4n-6/C18:2n-6 (D6D)*^1,2^*	0.22±0.07	0.26±0.10	<0.001
C16:1/C16:0 (D9D-16)*^3^*	0.03(0.02, 0.04)	0.04(0.03, 0.05)	<0.001
C18:1n-9/C18:0(D9D-18)*^1^*	1.65±0.39	1.84±0.43	<0.001
n-3/n-6^3^	0.08(0.06, 0.10)	0.08(0.06, 0.09)	0.176

*1*: Mean±SD

*2*: The data were logarithmically transformed.

*3*: Median (25 Percentiles, 75 Percentiles)

*4*: Adjusted for gender, age, BMI, BP, TC, TG, HDL-C, and LDL-C.

### Genotype distribution of five selected SNPs

The genotype distributions of the five SNPs were in Hardy-Weinberg Equilibrium, with p>0.05 in control subjects. Figure S2 shows the normalized melting curves and peaks of small amplicons. As shown in Table 4, logistic regression analysis revealed that rs174537 was associated with CAD in both additive model [OR = 0.548, 95% CI (0.385, 0.780), p = 0.001] and dominant model [OR = 0.732, 95% CI (0.555, 0.967), p = 0.028], rs174460 was also associated with CAD in both additive model [OR = 1.896, 95% CI(1.172, 3.067), p = 0.009] and dominant model [OR = 1.329, 95% CI (1.033, 1.711), p = 0.027]. The minor T allele of rs174537 was associated with a lower risk of CAD [OR = 0.743, 95% CI (0.624, 0.884), p = 0.001], while carriers of the rs174460 C allele were associated with a higher risk of CAD [OR = 1.357, 95% CI (1.106, 1.665), p = 0.003].

**Table 4:** Risk estimate based on the distributions of genotype and allele frequency.

SNP	genotype	Control (n = 510)	CAD (n = 505)	Allele OR(95% CI), value*^1^*	Additive OR(95% CI), P value*^2^*	Dominant OR(95% CI), P value*^2^*
rs174537G/T	GG	124	154	**0.743(0.624, 0.884), 0.001**	reference	reference
	GT	246	256		0.837(0.623, 1.124), 0.236	**0.732(0.555, 0.967), 0.028**
	TT	140	95		**0.548(0.385, 0.780), 0.001**	
rs174616C/T	CC	236	224	1.019(0.846, 1.228), 0.842	reference	reference
	TC	224	237		1.097(0.846, 1.422), 0.485	1.064 (0.830, 1.364), 0.625
	TT	50	44		0.916(0.587, 1.430), 0.700	
rs174611C/T	TT	500	501	0.402(0.126, 1.285), 0.112	reference	reference
	CT	10	4		0.376 (0.117, 1.210), 0.101	0.376 (0.117, 1.210), 0.101
rs174460C/T	TT	323	284	**1.357(1.106, 1.665), 0.003**	reference	reference
	TC	157	171		1.221(0.932, 1.600), 0.147	**1.329(1.033, 1.711), 0.027**
	CC	30	50		**1.896(1.172, 3.067), 0.009**	
rs174450A/C	AA	211	208	0.981(0.817, 1.177), 0.833	reference	reference
	AC	241	245		1.023(0.787, 1.330), 0.865	1.000(0.778, 1.286), 0.998
	CC	58	52		0.904(0.592, 1.380), 0.640	

*1*: P values derived from the chi-square test of allele frequency.

*2*: P values derived from logistic regression after adjustment for gender and age of genotype distribution.

**Figure 1:**
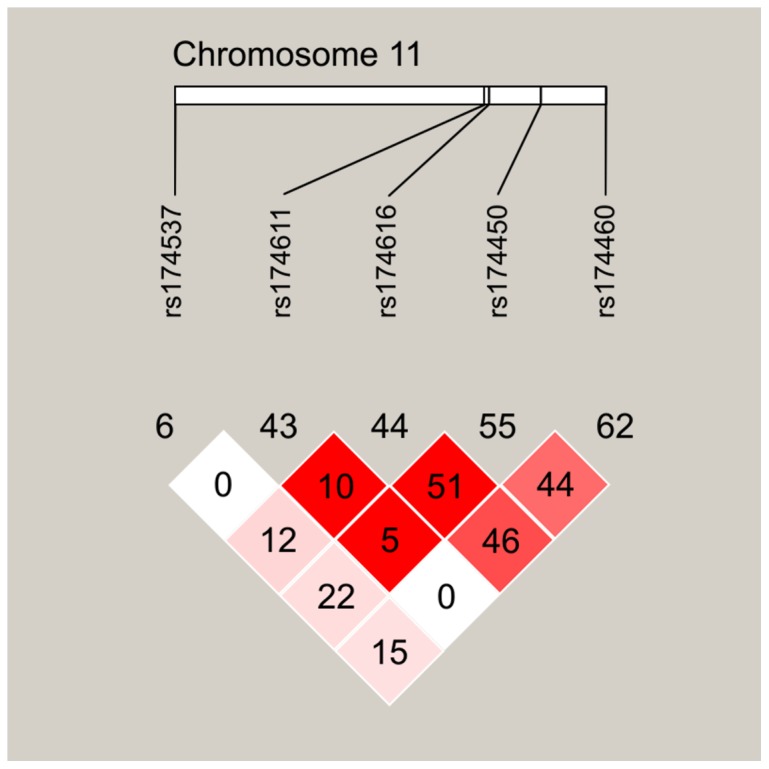
The schematic overview of linkage disequilibrium of the five studied SNPs (located on chromosome 11) . Color scheme represent D′/LOD, while the numbers stand for r^2^.

Linkage disequilibrium was performed with Haploview software (Figure 1). The SNP linkage disequilibrium patterns were assessed using both the D′ and r^2^ values. Based on the HapMap database, r^2^ was less than 0.8 among the five SNPs, suggesting that they do not exist in linkage disequilibrium with each other. Although rs174616 and rs174611 are adjacent to each other, no linkage disequilibrium was found between them.

### Plasma fatty acid levels, desaturase activity and SNPs

Among the five studied SNPs, rs174537 and rs174460 SNP distributions differed between the two groups. Thus, we further analyzed the effects of rs174537 SNP (Table 5) and rs174460 SNP (Table 6) on lipids and plasma fatty acid levels. There were significant differences among different genotype groups in lipids and fasting plasma glucose (FPG). All fatty acids differed among different genotypes in both rs174537 and rs174460, with the exception of C18:0 and AA.

**Table 5:** Effects of rs174537 SNP on lipids and plasma fatty acid levels.

Characteristics	Controls		CAD patients	
	GG(n = 124)	GT+TT(n = 386)	GG(n = 154)	GT+TT(n = 351)
TC (mmol/l)*^1^*	4.43(4.05, 4.80)	4.32(3.74, 4.77)^△^	3.86(3.20, 4.42)*^,&^	4.27(3.83, 5.14)^□,§^
TG (mmol/l)*^1^*	1.01(0.79, 1.37)	1.02(0.77, 1.34)	1.17(0.89, 1.66)*^,&^	1.44(1.03, 1.76)^□,§,#^
HDL-C (mmol/l)*^1^*	1.36(1.17, 1.58)	1.25(1.04, 1.48)^△^	1.11(0.93, 1.33)*^,&^	1.17(1.03, 1.37)^§,#^
LDL-C (mmol/l)*^1,2^*	2.70±0.44	2.57±0.63^△^	2.14(1.58, 2.65)*^,&^	2.52(2.22, 3.23)^□^
FPG (mmol/l)*^1^*	5.04(4.76, 5.32)	4.93(4.57, 5.43)	6.05(5.42, 6.81)*^,&^	5.54(5.09, 6.29)^□,§,#^
Palmitic acid, C16:0*^2,3^*	22.28±4.48	22.28±3.76	22.89±3.38	23.30±2.38^□,§,#^
Palmitoleic acid, C16:1*^1^*	0.62(0.45, 0.90)	0.66(0.45, 0.92)	0.97(0.74, 1.28)*^,&^	0.96(0.69, 1.24)^§,#^
Stearic acid, C18:0*^2,3^*	8.73±2.39	9.02±2.01	9.30±1.42	9.21±1.11
Oleic acid, C18:1n-9*^2^*	14.79±3.44	14.70±3.27	16.15±2.75*^,&^	16.56±2.80^#,§^
Linoleic acid, C18:2n-6*^1,2^*	35.04(30.21, 39.44)	36.64(32.81, 40.13)^△^	33.06±4.90*^,&^	33.75±4.27^#,§^
γ-linolenic acid, C18:3n-6*^1^*	0.19(0.00, 0.81)	0.16(0.00, 0.43)	0.33(0.20, 0.50)*^,&^	0.29(0.11, 0.47)^□,§^
α -linolenic acid, C18:3n-3*^1^*	0.43(0.24, 0.70)	0.44(0.29, 0.68)	0.68(0.52, 0.91)*^,&^	0.63(0.39, 0.82)^□,§,#^
Dihomo-γ-linolenic acid, C20:3n-6*^1^*	1.37(0.96, 1.90)	1.29(0.97, 1.69)	1.63(1.26, 1.91)*^,&^	1.50(1.19, 1.89)^§^
Arachidonic acid, C20:4n-6*^2^*	7.78±2.64	7.86±2.47	7.84±2.18	8.03±2.13
Eicosapentaenoic acid, C20:5n-3*^1^*	0.06(0.00, 0.29)	0.10(0.00, 0.30)	0.37(0.18, 0.54)*^,&^	0.23(0.02, 0.45)^□,§,#^
Docosahexaenoic acid, C22:6n-3*^2^*	2.62±1.07	2.80±1.06	2.51±0.68^&^	2.60±0.76^§^
C20:4n-6/C20:3n-6 (D5D)*^1^*	6.35(3.67, 9.01)	6.54(4.42, 8.50)	4.65(3.70, 6.75)*^,&^	5.38(3.88, 7.36)^□,§^
C20:4n-6/ C18:2n-6 (D6D)*^2,3^*	0.23±0.08	0.23±0.08	0.25±0.10^&^	0.25±0.09^#,§^
C16:1/ C16:0 (D9D-16)*^1^*	0.03(0.02, 0.04)	0.03(0.02, 0.04)	0.04(0.03, 0.06)*^,&^	0.04(0.03, 0.05)^§,#^
C18:1n-9/ C18:0(D9D-18)*^2^*	1.76±0.43	1.67±0.42^△^	1.76±0.38^&^	1.82±0.42^§^

*1*: Median (25 Percentiles, 75 Percentiles).

*2*: Mean±SD.

*3*: The data were logarithmically transformed.

△: Control-GG vs Control-GT+TT, *: Control-GG vs CAD-GG, #: Control-GG vs CAD-GT+TT

&: Control-GT+TT vs CAD-GG, §: Control-GT+TT vs CAD-GT+TT, □: CAD-GG vs CAD-GT+TT

**Table 6:** Effects of rs174460 SNP on lipids and plasma fatty acid levels.

Characteristics	Controls		CAD patients	
	TT(n = 323)	CC+CT(n = 187)	TT(n = 284)	CC+CT(n = 221)
TC(mmol/l)*^1^*	4.38(3.91, 4.77)	4.17(3.58, 4.82)^△^	4.03(3.31, 4.67)*	4.44(3.98, 5.19)^□,§^
TG(mmol/l)*^1^*	0.98(0.77, 1.34)	1.05(0.80, 1.43)	1.23(0.93, 1.71)*^,&^	1.47(1.04, 1.77)^□,§,#^
HDL-C (mmol/l)*^1^*	1.34(1.14, 1.56)	1.17(0.96, 1.39)^△^	1.11(0.92, 1.32)*	1.17(1.08, 1.40)^□,#^
LDL-C(mmol/l)*^1,2^*	2.69±0.46	2.44±0.74^△^	2.39(1.73, 2.82)*	2.58(2.32, 3.26)^□,§^
FPG (mmol/l)*^1^*	4.91(4.60, 5.22)	5.19(4.63, 5.93)^△^	5.92(5.26, 6.42)*^,&^	5.47(4.99, 6.22)^□,§,#^
Palmitic acid, C16:0*^2,3^*	22.58±3.87	21.76±4.02	23.21±3.24^&^	23.13±1.87^§^
Palmitoleic acid, C16:1*^1^*	0.61(0.44, 0.91)	0.71(0.49, 0.93)	0.96(0.70, 1.25)*^,&^	0.96(0.71, 1.26)^§,#^
Stearic acid, C18:0*^2,3^*	9.09±2.03	8.72±2.23	9.26±1.34	9.19±1.05
Oleic acid, C18:1n-9*^2^*	14.76±3.32	14.64±3.30	16.31±2.72*^,&^	16.44±2.70^#,§^
Linoleic acid, C18:2n-6*^2,3^*	36.83(32.86, 40.79)	35.67(30.41, 39.11)^△^	33.51±4.80^&^	33.57±4.04^§,#^
γ-linolenic acid, C18:3n-6*^1^*	0.14 (0.00, 0.39)	0.21(0.01, 0.60)	0.31(0.16, 0.50)*	0.29(0.13, 0.45)^#^
α -linolenic acid, C18:3n-3*^1^*	0.45(0.29, 0.70)	0.43(0.27, 0.65)	0.64(0.46, 0.84)*^,&^	0.66(0.41, 0.86)^§,#^
Dihomo-γ-linolenic acid, C20:3n-6*^1^*	1.26(0.95, 1.71)	1.36(1.00, 1.81)	1.52(1.21, 1.84)*^,&^	1.54(1.20, 1.96)^§,#^
Arachidonic acid, C20:4n-6*^2^*	7.89±2.46	7.74±2.60	8.10±2.34	7.89±1.91
Eicosapentaenoic acid, C20:5n-3*^1^*	0.09(0.00, 0.29)	0.10(0.00, 0.30)	0.27(0.00, 0.46)*^,&^	0.30(0.00, 0.52)^§,#^
Docosahexaenoic acid, C22:6n-3*^2^*	2.84±1.04	2.62±1.09	2.63±0.78*	2.52±0.69^#^
C20:4n-6/C20:3n-6 (D5D)*^1^*	6.65(4.49, 8.56)	6.31(3.97, 8.65)	5.12(3.82, 7.65)*	5.20(3.80, 6.79)^§,#^
C20:4n-6/C18:2n-6 (D6D)*^2^*	0.22±0.08	0.24±0.09	0.25±0.10*	0.25±0.08^#^
C16:1/C16:0 (D9D-16)*^1^*	0.03(0. 02, 0.04)	0.03(0.02, 0.04)^△^	0.04(0.03, 0.05)*^,&^	0.04(0.03, 0.05)^§,#^
C18:1n-9/C18:0 (D9D-18)*^2^*	1.65±0.41	1.75±0.43^△^	1.79±0.41*	1.81±0.41^#^

*1*: Median (25 Percentiles, 75 Percentiles).

*2*: Mean±SD.

*3*: The data were logarithmically transformed.

△: Control-TT vs Control-CC+CT, *: Control-TT vs CAD-TT, #:Control-TT vs CAD-CC+CT.

&: Control-CC+CT vs CAD-TT, §:Control-CC+CT vs CAD-CC+CT, □: CAD-TT vs CAD-CC+CT.

Compared with controls of rs174537 GG genotype, CAD patients of rs174537 GG genotype had lower D5D and higher D9D-16; CAD patients of rs174537 GT+TT genotype had higher D6D and D9D-16. Compared with controls of rs174537 GT+TT genotype, CAD patients of rs174537 GG genotype had decreased D5D and increased D6D, D9D-16, D9D-18; CAD patients of rs174537 GT+TT genotype showed reduced D5D, and elevated D6D, D9D-16, D9D-18. CAD patients of rs174537 GG genotype had lower D5D than GT+TT genotype patients.

Compared with controls of rs174460 TT genotype, controls of rs174460 CT+CC genotype had higher D9D-16 and D9D-18; lower D5D and higher D6D, D9D-16, D9D-18 were found in all patients. Compared with controls of rs174460 CT+CC genotype, CAD patients of rs174460 TT genotype had increased D9D-16; CAD patients of rs174460 CT+CC genotype had decreased D5D and increased D9D-16.

## Discussion

In this paper, we used the high-resolution melting to analysis FADS gene cluster polymorphisms with the plasma level of fatty acids in 510 healthy individuals and 505 CAD patients. And for the first time, the rs174460 is reported to be associated with CAD risk.

Our study found that three desaturase activities (D9D, D5D and D6D) were associated with CAD in a Chinese Han population. The results showed that the fatty acid composition in plasma and the estimated desaturase activities were significantly different between controls and CAD patients. SCD activities, both D9D-16 and D9D-18, were significantly higher in patients with CAD than control subjects, and the main product, C16:0, was also increased. This result supports a previous report that high SCD activity is an independent predictor of cardiovascular risk factors [6]. Studies by Sampat [16] and Lelliott [17] suggested that high SCD activity may be associated with increased lipogenesis and influence ectopic fat deposition and thereby insulin resistance via lipotoxic mechanisms.

CAD patients had lower level of LA than the control group. This result may be in agreement with the report of Warensjö [6]: LA was a major influencing factor on arterial stiffness. Potentially, sufficient amounts of LA in the serum or diet could improve insulin sensitivity and reduce coronary heart disease risk or mortality [18], [19]. Petersson et al. [20] also found that higher plasma LA was associated with lower inflammation and lower cardiovascular risk. AA as the direct precursor of strong inflammatory eicosanoids (such as PGs, LTs and lipoxins), is thought to be an important factor for the development of some complex diseases. In the present study, AA was significantly higher in CAD patients (p<0.01). As stated above, this increase may be one of the reasons for the formation of plaques in atherosclerosis. Therefore, D6D activity, presented as AA/LA, was also higher in CAD patients (p<0.001). Martinelli et al. [15] demonstrated that a higher AA/LA ratio was an independent risk factor for CAD in a multiple logistic regression model. This is consistent with our result of higher D6D activity.

In addition, we observed high DHA level in controls, which is consistent with the established cardiovascular protective effect of increased n-3 PUFA exposure [21]. However the protect mechanisms of DHA is still not clear.

We established genotyping methods of five SNPs in the FADS gene cluster by high-resolution melting and successfully used it in 1015 samples. The results showed that the genotype distributions of rs174537 G>T and rs174460 C>T were different between the CAD and control group after adjustment.

For the rs174537 SNP, T allele carrier of controls had a lower level of D9D-18. While G allele carrier of CAD patients showed increased D6D, D9D-16, D9D-18, and decreased D5D. Our data demonstrated that the rs174537 T allele was associated with a lower risk of CAD [OR 0.743, 95% CI (0.624, 0.884), p = 0.001]. This result is consistent with the report of Jung Hyun in Korea [22]. And a possible protective effect of increased D5D activity on coronary heart disease may partly be mediated by increased plasma level of DHA.

Rs174537 is located in an intron and is adjacent to the FADS1 gene. Recently, several studies have reported that rs174537 is in linkage disequilibrium with rs174546 (r^2^ = 0.99) and is associated with expression of FADS1 in lymphoblastoid cells [23]. Therefore, this variant may be a marker of other functional polymorphisms or in linkage with other variants affecting fatty acid concentrations and, consequently, CAD.

For the rs174460 SNP, C allele carriers, including controls and patients, had higher levels of D6D, D9D-16, D9D-18, and lower level of D5D. Our findings suggest that the rs174460 C allele was associated with a higher risk of CAD [OR 1.357, 95% CI (1.106, 1.665), p = 0.003].

Rs174460 is located in the FADS3 gene. The FADS3 gene function is still unknown; however, it is presumed to have desaturase activity because of its sequence homology with FADS1 and FADS2 genes (62% and 70% nucleotide sequence identity, respectively) [24]. Malerba G [25] also showed a significant correlation between FADS3 polymorphism (rs1000778) and PUFAs.

In the present study, carriers of rs174460 C allele had a higher level of DGLA. In an earlier report, Kim OY [3] showed a similar result in individuals with more features of metabolic syndrome and arterial stiffness. They proposed that impaired fatty acids metabolism may cause the accumulation of DGLA, possibly as a consequence of long-term metabolic disorders. This theory was based on reports by Warensjö et al. [6] and others [3]: LA and α -linolenic acid (ALA, C18:3n-3) in plasma lipid esters reflect the dietary fatty acid intake 6–8 weeks before measurement. However, C16:1 and DGLA do not reflect the dietary intake of those fatty acids but are synthesized endogenously by a series of desaturation step.

Several studies, including genome-wide association studies, showed associations between SNPs in the FADS gene cluster and lipid levels, such as HDL-C, LDL-C and TG [26], [27]. Although our study also showed significant difference in lipid levels with SNPs between the two groups, the results were not fully consistent with previous reports, such as TC and LDL-C were decreased in CAD patients. The main reason was the effect of drugs or treatments. Many patients with CAD also suffer from hyperlipidemia and take lipid-lowering drugs (mainly stains in our CAD patients) and do not take n-3 PUFAs or fish oil, which may partly explain these results. Statins are inhibitors of hydroxymethyl glutaryl coenzyme A (HMG-CoA) reductase. These drugs inhibit endogenous HMG-CoA reductase by competition and blocking the mevalonate metabolic pathway in cells, increasing the clearance of serum cholesterol. Therefore, the results do not truly reflect the situation of lipids in CAD patients.

Besides, no significant difference was found in n-3/n-6 between controls and CAD patients.

Our study had several limitations. First, no SNPs were evaluated in the SCD gene; thus there was no information about the association of the SCD gene polymorphism with the composition of plasma fatty acids. Second, the concentrations of plasma fatty acids are influenced by both dietary intake and metabolic pathways. However, we did not obtain any information about energy intake.

Overall, we firstly report that the rs174460 C allele is associated with a higher risk of CAD, and confirm that the rs174537 T allele is associated with a lower risk of CAD. Our results indicate that FADS gene polymorphisms are likely to influence plasma fatty acid concentrations and desaturase activities. Further investigations are needed to explore the potential mechanisms of rs174460 C allele and increased D6D, D9D activities and higher CAD risk.

## Supporting Information

Figure S1 **Representative Chromatograms of plasma fatty acids by gas chromatography.**
(DOC)

Figure S2 **High-resolution melting curves of five studied SNPs.**
(DOC)

Table S1 Amplification primers utilized in the genotype.
(DOC)
