# Supplementary material for: FADS Gene Polymorphisms Confer the Risk of Coronary Artery Disease in a Chinese Han Population through the Altered Desaturase Activities: Based on High-Resolution Melting Analysis
Source: PLoS One. 2013 Jan 31;8(1):e55869. doi: 10.1371/journal.pone.0055869 (PMC3561316; doi:10.1371/journal.pone.0055869)
Supplement: Figure S1 — Representative Chromatograms of plasma fatty acids by gas chromatography. (DOC) [file pone.0055869.s001.doc]

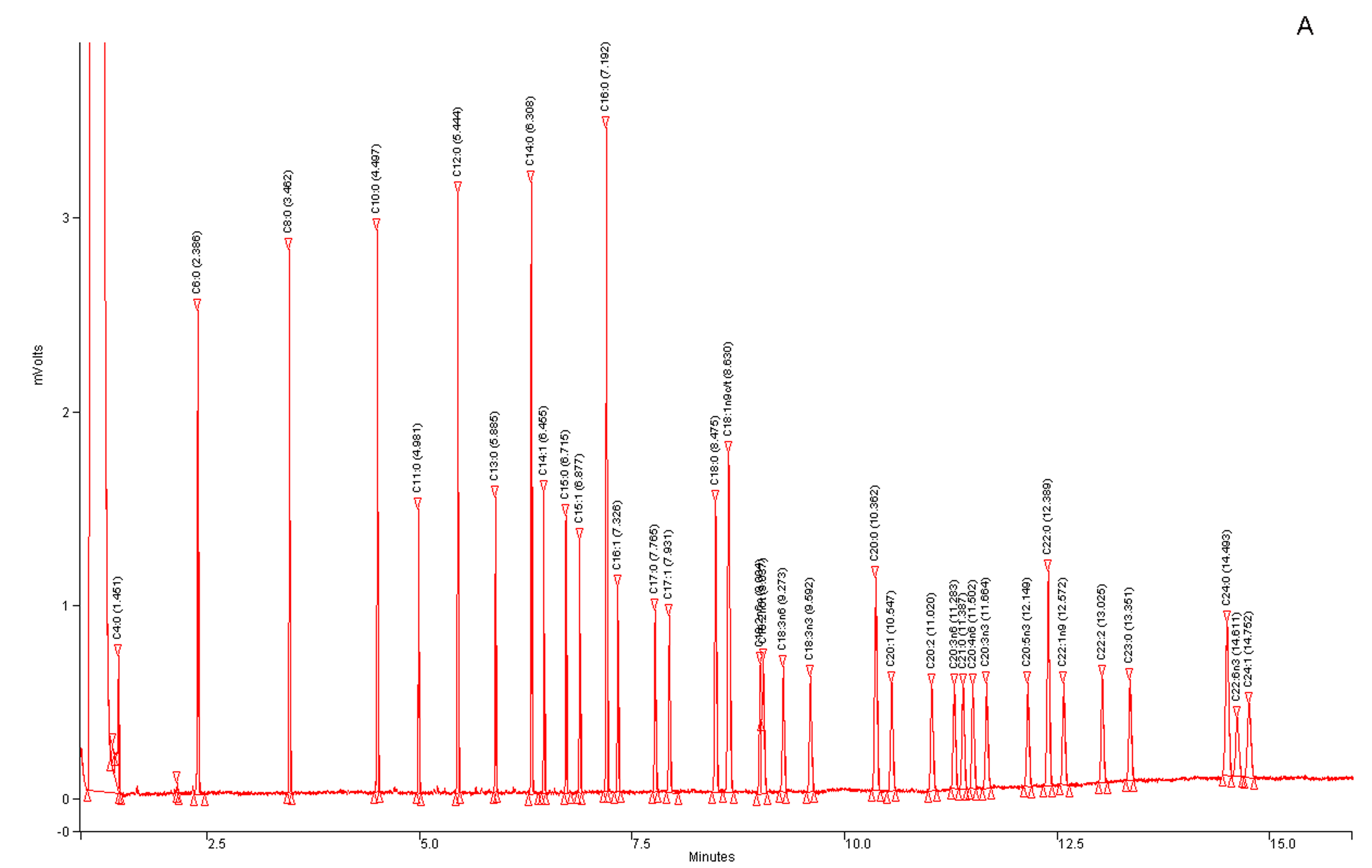


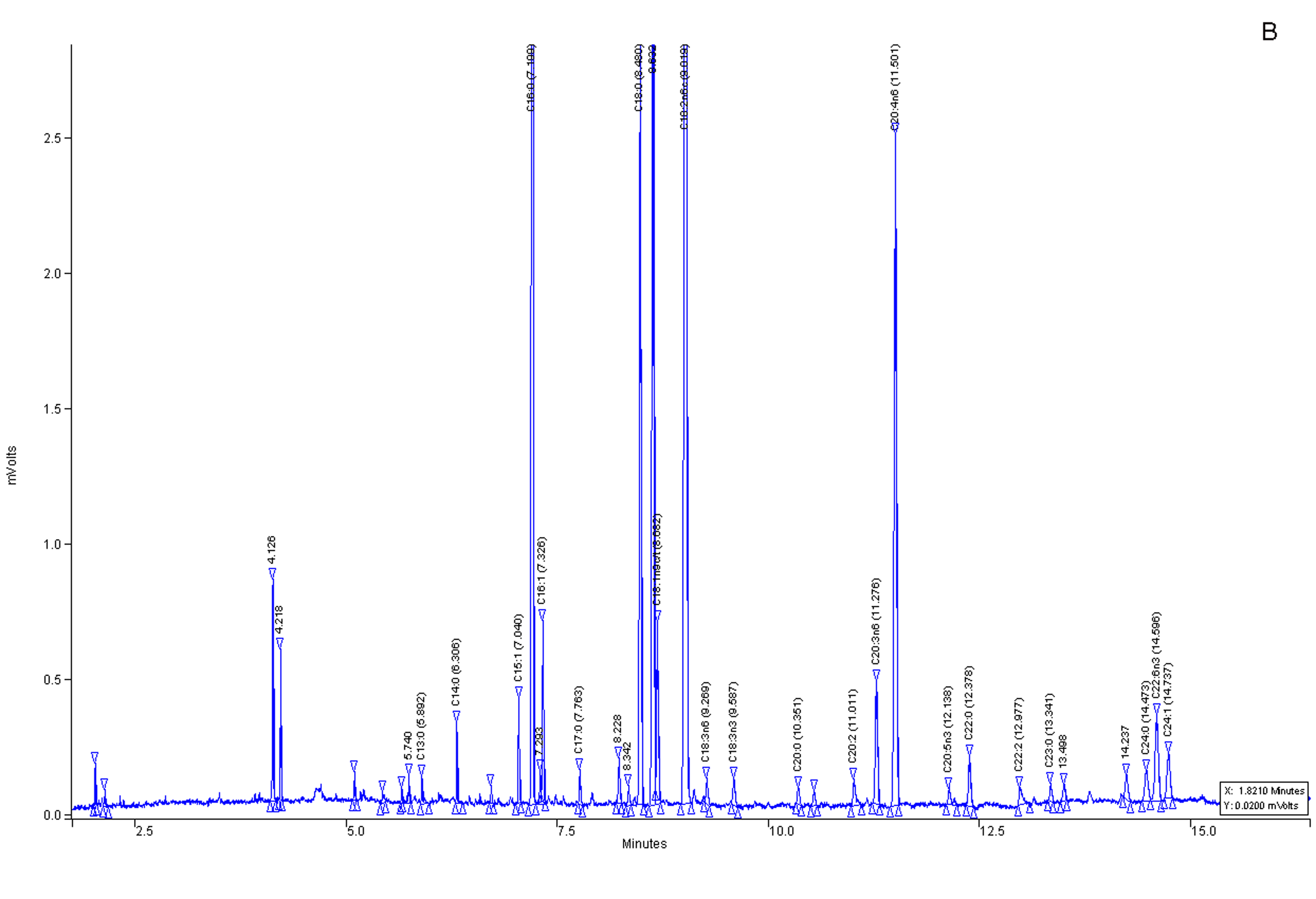

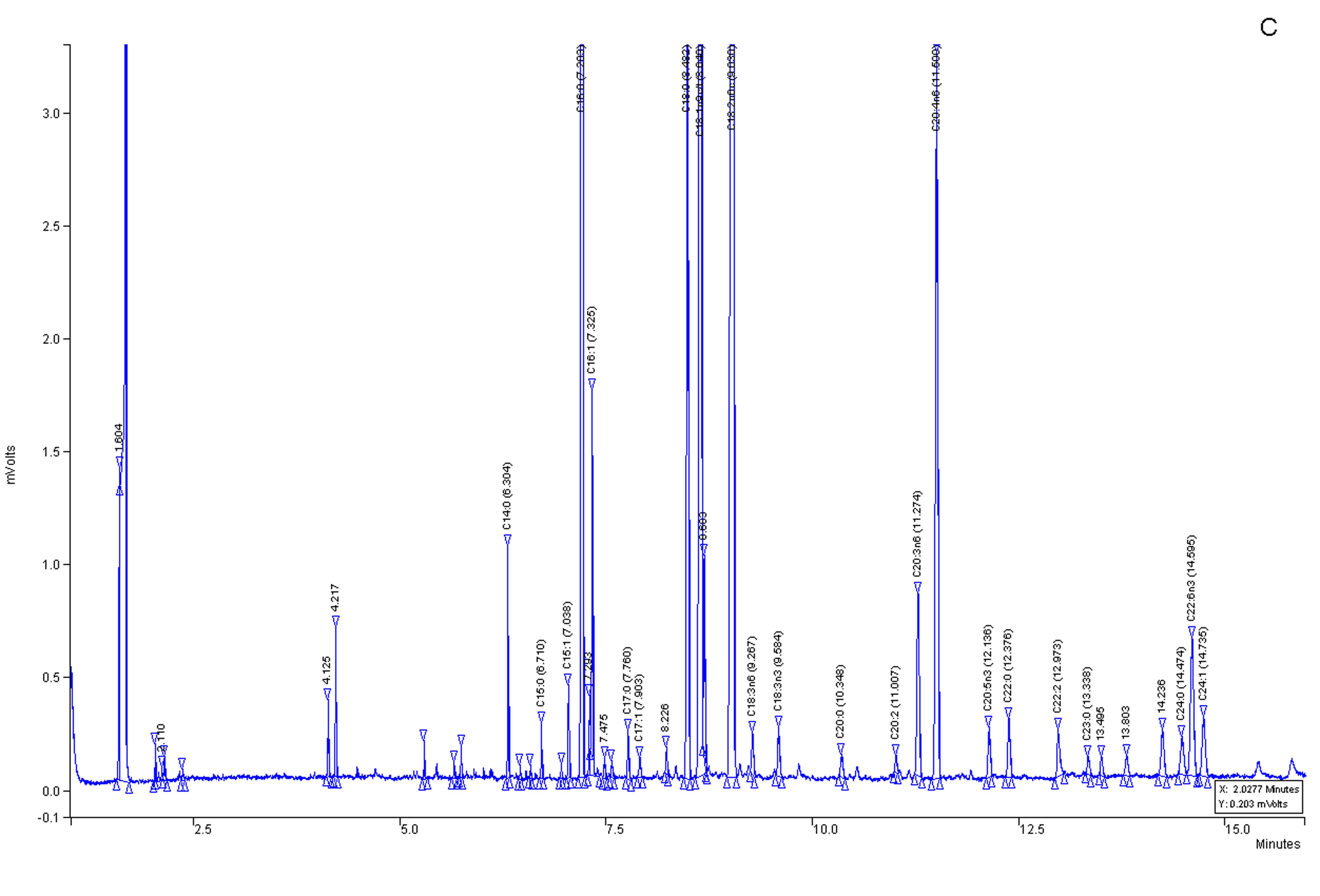


Figure S1 Representative Chromatograms of plasma fatty acids by gas chromatography

A: fatty acid methyl ester standards; B: plasma fatty acid methyl ester of a patient with coronary artery disease; C: plasma fatty acid methyl ester of a control.
