# Supplementary material for: FADS Gene Polymorphisms Confer the Risk of Coronary Artery Disease in a Chinese Han Population through the Altered Desaturase Activities: Based on High-Resolution Melting Analysis
Source: PLoS One. 2013 Jan 31;8(1):e55869. doi: 10.1371/journal.pone.0055869 (PMC3561316; doi:10.1371/journal.pone.0055869)
Supplement: Figure S2 — High-resolution melting curves of five studied SNPs. (DOC) [file pone.0055869.s002.doc]

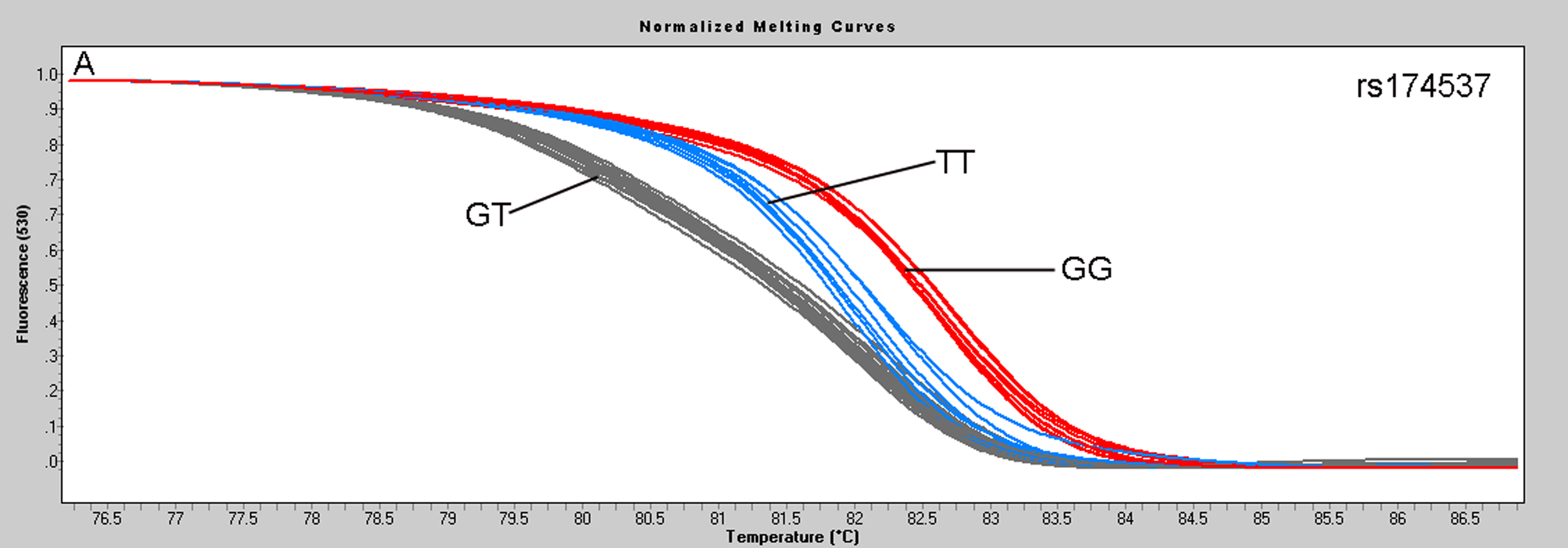

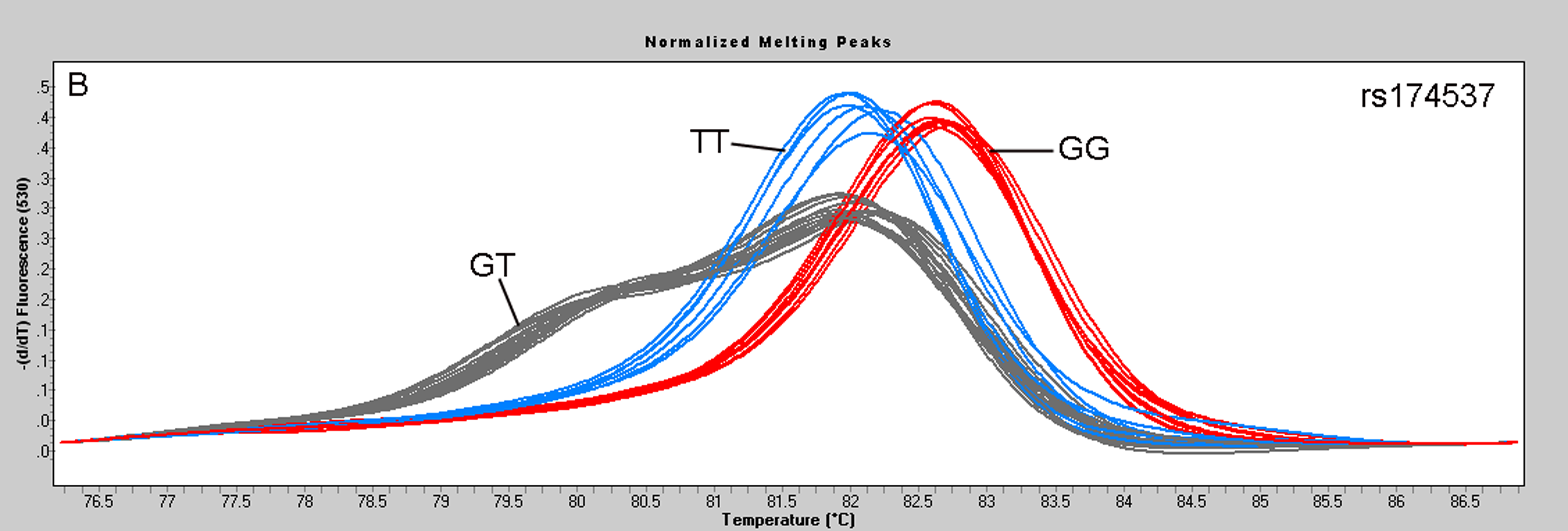

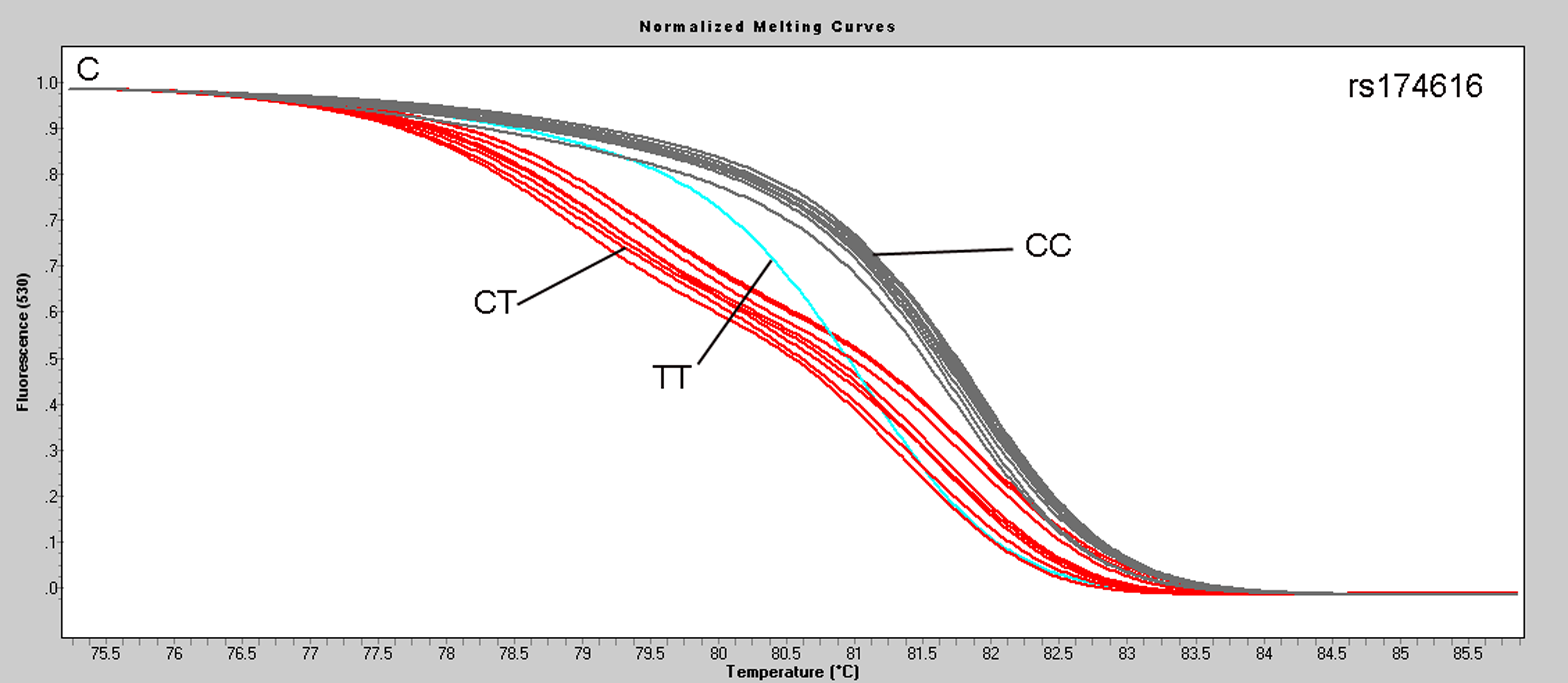

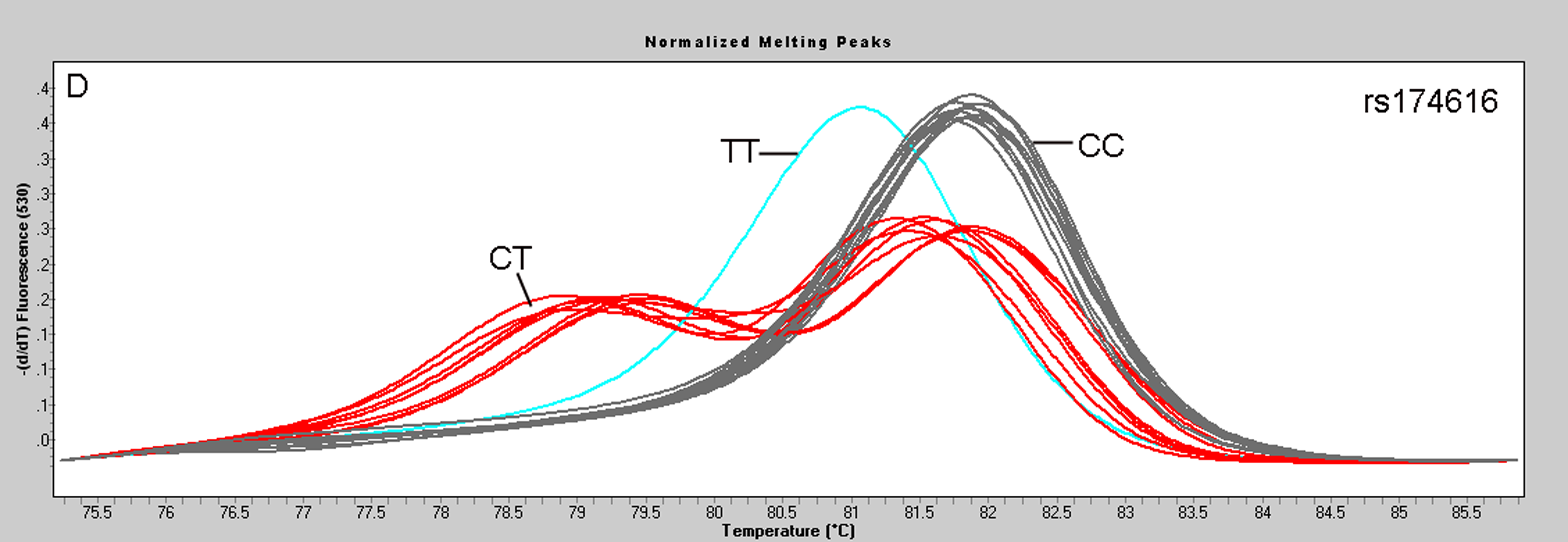

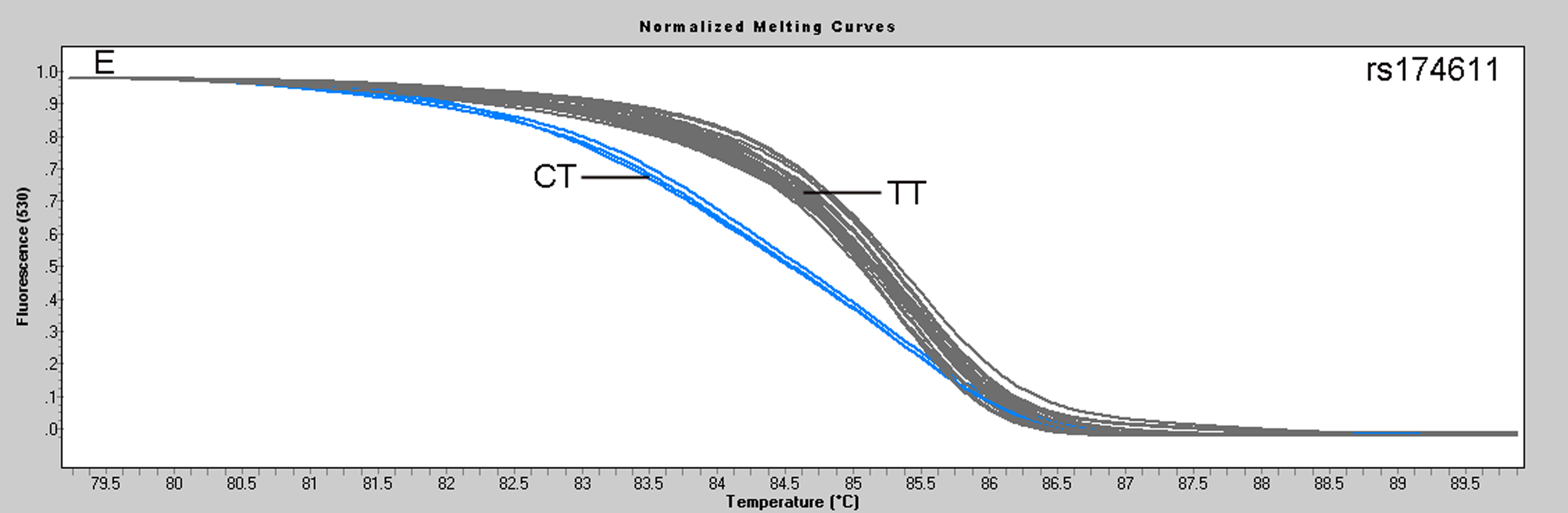

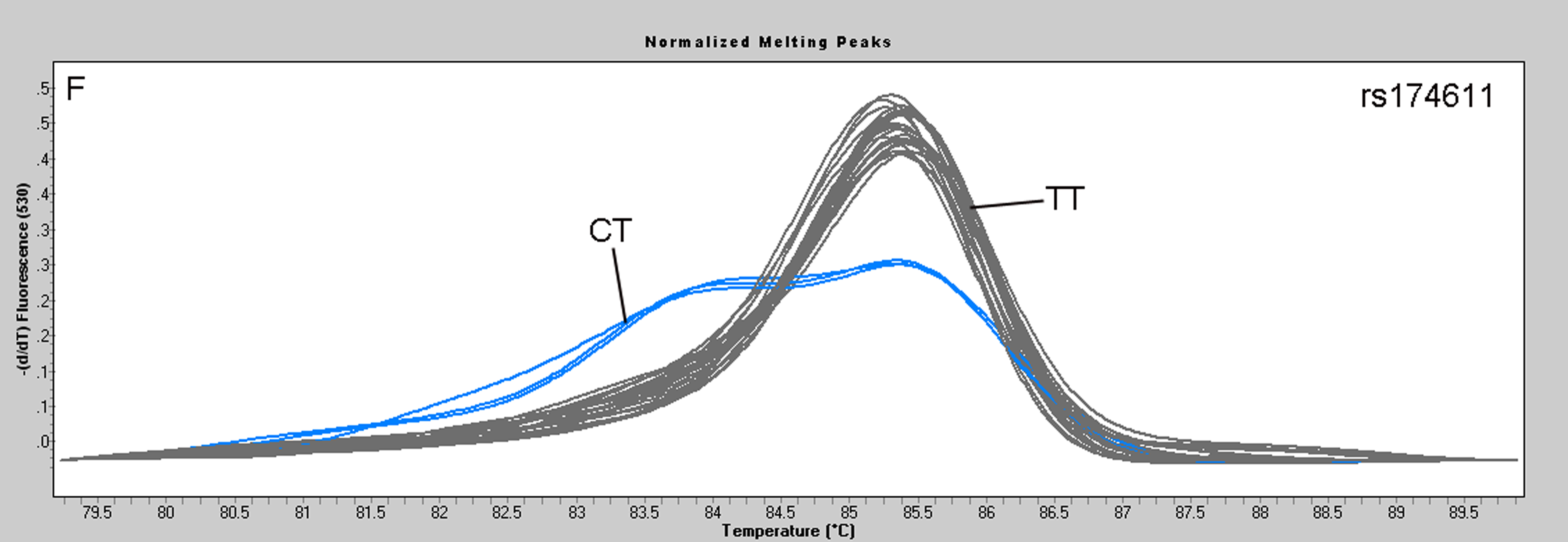

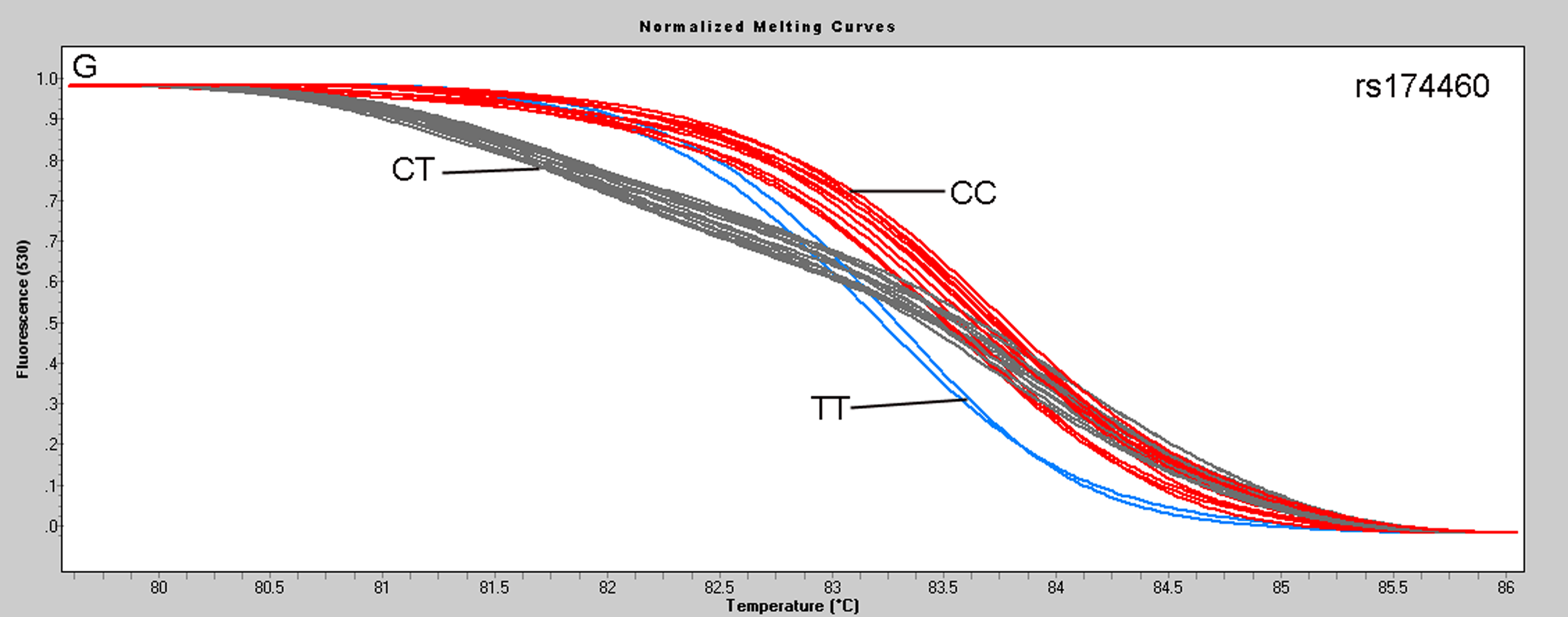

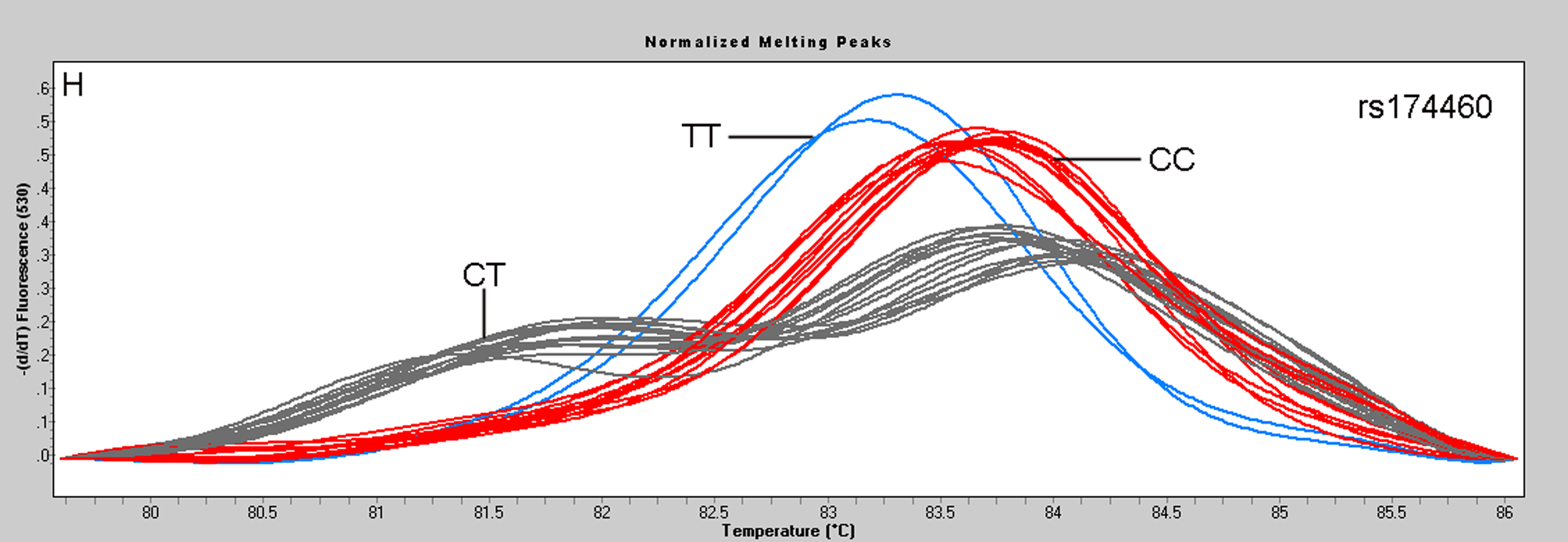

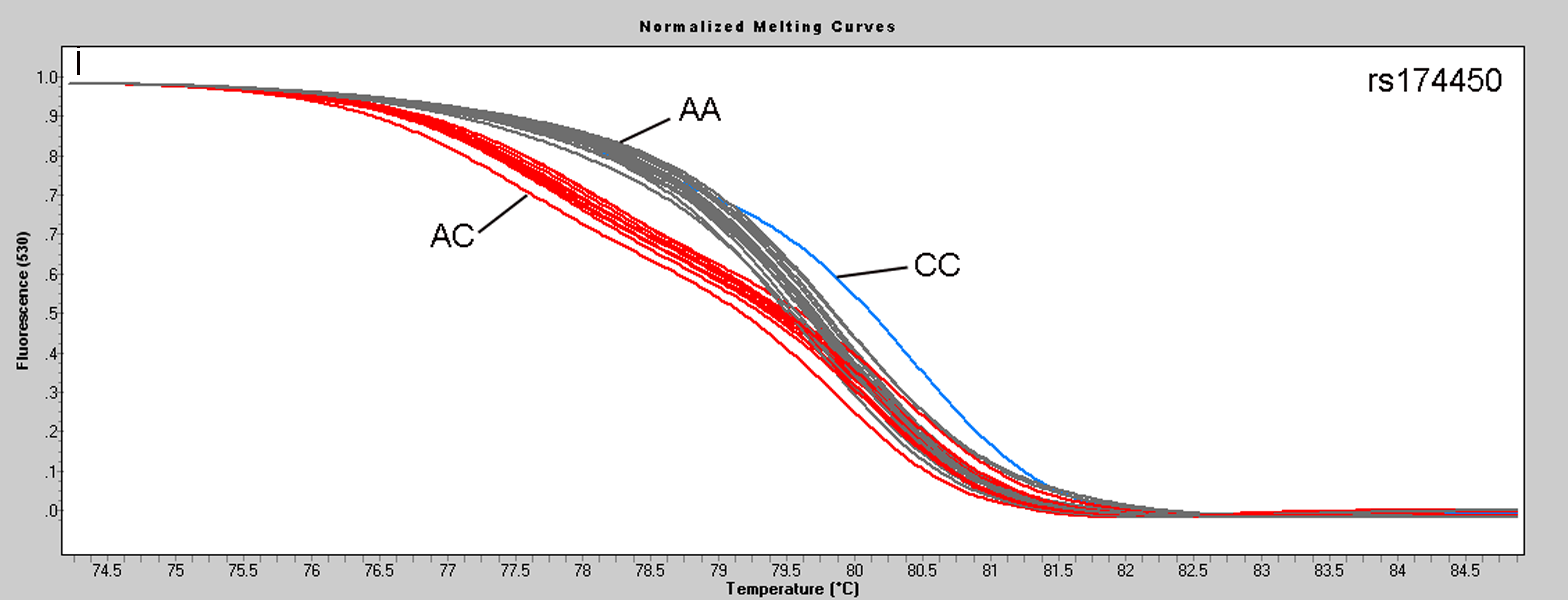


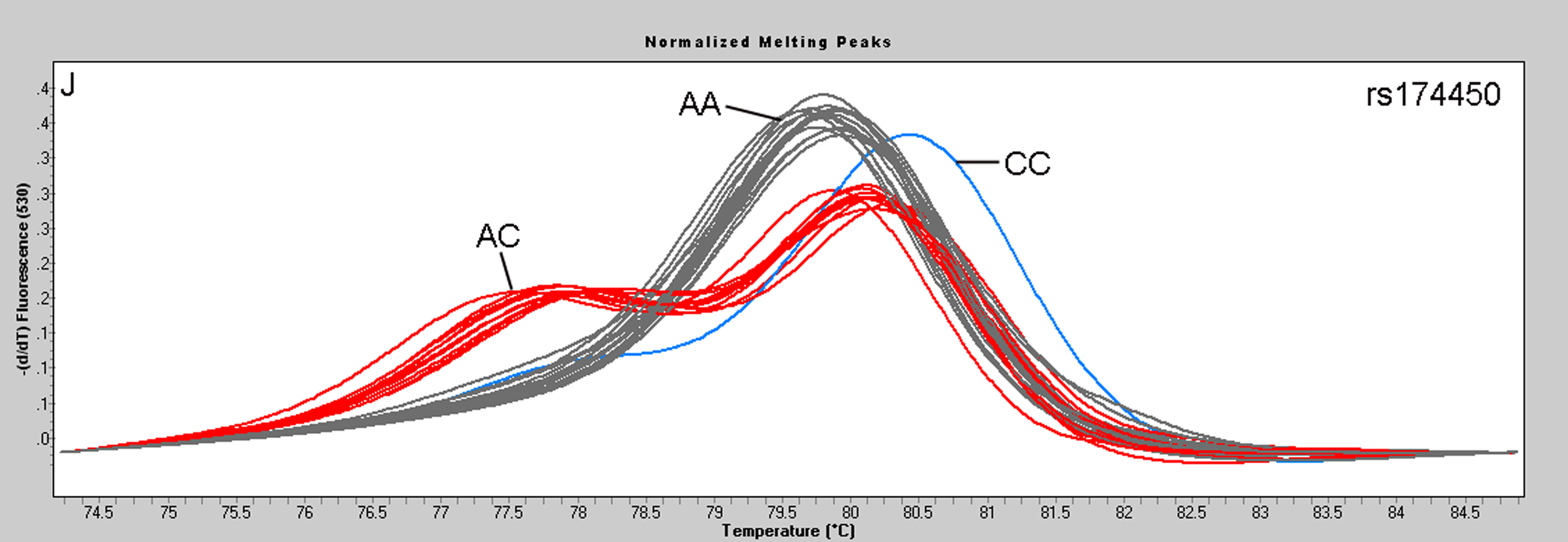


Figure S2 High-resolution melting curves of five studied SNPs.

A, C, E, G, I: normalized melting curves of rs174537, 174616, rs174611, 174460, 174450, respectively.

B, D, F, H, J: normalized melting peaks of rs174537, 174616, rs174611, 174460, 174450, respectively.
