# Supplementary material for: FADS Gene Polymorphisms Confer the Risk of Coronary Artery Disease in a Chinese Han Population through the Altered Desaturase Activities: Based on High-Resolution Melting Analysis
Source: PLoS One. 2013 Jan 31;8(1):e55869. doi: 10.1371/journal.pone.0055869 (PMC3561316; doi:10.1371/journal.pone.0055869)
Supplement: Table S1 — Amplification primers utilized in the genotype. (DOC) [file pone.0055869.s003.doc]

Table S1 Amplification primers utilized in the genotype

| SNP | Primers(5’→3’) | product length(bp) | Tm(℃) |
| --- | --- | --- | --- |
| rs174537Forward | CCCTGTCGCCCTGCAGAA | 50 | 61 |
| Reverse | CTGGGCTCTCCCTCTGTCTTG |  |  |
| rs174616Forward | TTGGCCGAGGGCAGGAC | 50 | 65 |
| Reverse | CTCACCTTGAAGGCCACCTTATTG |  |  |
| rs174611Forward | TCTGGCAAGGTTCCCTGTT | 62 | 61 |
| Reverse | GTGAAGCGTGGGAGCATC |  |  |
| rs174460Forward | CGCCATTGCACTCCAGTC | 57 | 55 |
| Reverse | CCACCAACCGCCGGAGA |  |  |
| rs174450Forward | CTACCCGACACCCGTCA | 50 | 61 |
| Reverse | TGCTCATTCTCTGGAATGGAATCT |  |  |
